# Living history, fading minds: dementia as the gateway to historical past in South Africa via Antjie Krog’s *Blood’s Inner Rhyme* (2025) and Julia Martin’s *The Blackridge House* (2019)

**DOI:** 10.1093/geront/gnag221

**Published:** 2026-09-12

**Authors:** Oscar Ortega Montero

**Affiliations:** Wits Institute for Social and Economic Research (WiSER), Wits University, Johannesburg, South Africa

**Keywords:** Dementia, South Africa, Life writing, Aging studies

## Abstract

This article analyzes the literary, social, and cultural representations of dementia in Antjie Krog’s *Blood’s Inner Rhyme* and Julia Martin’s *The Blackridge House*. I contend that the authors’ accounts of their mothers’ struggle with dementia, from its onset to its end, function as living archives of personal and national history. I employ life writing, aging studies, and, to a lesser extent, historiography to examine the literary, social-cultural, and historical dimensions of such experiences. I address the narrative construction of the mothers’ identity in later life by highlighting the role of archives and family memories in the context of contemporary South Africa. Thus, I aim to illustrate the instrumental role of life narratives in the creation of safe spaces for intergenerational dialogue and reconciliation with a troubled past on a personal and national level. I also contend that humanities-based inquiry brings to the fore the essential aspects of later life, thereby challenging the pathologizing medical in the representation of dementia, which often disregards the reality of old people as vessels of knowledge. Eventually, I showcase that illness life narratives function as accessible repositories of public knowledge that render visible the realities of aging.

Dementia can bring historical conflicts and wounds to the surface in unexpected ways. Very often, people living with dementia relive past events. This implies they continue to act out for as long as they think their behavior will eventually change the outcome of whatever troubles the person living with this condition. A handful of studies (Agarwal & Jhajharia, 2022; Craftman et al., 2020; Ione, 2023, 2024; Lewington et al., 2024) suggest that unmet demands, together with past conflicts and distressing events, contribute to increased risk of developing dementia because of their long-lasting psychological effects. In the context of South Africa, the legacies of colonialism, apartheid, and the so-called South African or Border War are the roots of the pervasive effects of historical conflict (Breckenridge, 2005; Chari, 2024; Simpson, 2022). All these historical conflicts combined may well have contributed to the risk and severity of dementia in older people, who have first-hand knowledge of the complexities surrounding the social, cultural, historical, and political specificities of South Africa. As a result, the processes accelerating both cognitive impairment and stress-related aging have been further accentuated.

Taking all this in mind, I examine dementia in connection with the manifestation of historical conflict and political scars in South Africa in old age through the analysis of two dementia life narratives, as I label them, namely Antjie Krog’s *Blood’s Inner Rhyme* (2025) and Julia Martin’s *The Blackridge House* (2019). In these powerful life narratives, Krog and Martin offer a candid yet painful account of their mothers’ journey through dementia, from its onset to its end. In the narratives, caregiving contexts promote dementia-friendly environments where the authors’ mothers’ longings emerge in the face of vulnerability. As the narratives attest, the burden of the past impacts Krog and Martin, who find shelter in the therapeutic benefits of life writing to confront a cycle of distress that persists over time. The life narratives under scrutiny, besides capturing and representing the human component of illness, facilitate and enrich our comprehension of ourselves and others (Polkinghorne, 2013, p. 23).

Furthermore, delving into both the historical past and our roots through the construction of life narratives, besides expanding self-understanding and understanding of our beloved ones, operates as an “ethical enquiry” (Meretoja, 2018, p. 90). Such ethical inquiry promotes opportunities for intergenerational dialogue. And more importantly, the narratives also mirror the possibility of healing on a national level. The healing component of the narratives, namely a somewhat fractious mother-daughter relationship on account of politics in the case of Krog and the discovery of Martin’s forebear’s involvement in the colonial construction of South Africa, can be extrapolated to the social climate in present-day South Africa, where tensions between the different social groups still persist. The narratives thus show that healing requires the navigation of relational and experiential aspects.

Historical conflict and political scars are intergenerational (Starrs & Békés, 2025). This fact is made evident in the narratives, as Serfontein’s and Betty’s lived experiences impact Krog and Martin in various ways, testing family resiliency in their bid to overcome the intricacies that are specific to post-conflict societies. This is the case of contemporary South Africa, where addressing the root causes of conflict while simultaneously building the foundations for inclusiveness is a must, as the narratives illustrate through the accounts of Serfontein and Betty. Contemporary South Africa is caught in the challenges of a transition to a just society after gaining political freedom (Nuttall, 2009). This implies the examination of deep cleavages, justice dilemmas, or the state’s capacity to build institutions that are held accountable. In the ideological-text world of *Blood’s Inner Rhyme* and *The Blackridge House*, memories of colonialism and the strict segregation laws under apartheid play a big part in the transmission of fear, conflict, and trauma (Maylan, 2016). Writing about those experiences helps Krog and Martin move past intergenerational wounds and start healing in the present.

Coping with wounds reads as a consequence of the deeply entrenched structural violence in today’s South Africa. The latter is primarily the result of racism and colonialism, which, again, have persisted over time. This scenario precipitates the search for some form of emotional reparation and restoration of culture, which is one of the driving forces of the stories. In varying degrees, the mothers appear to be stuck in the aftermath of the conflict and in the memories of the past, which is an aspect further exacerbated by the fact that both women battle with dementia. These two components derive from the development of a culture of enforced peace in a society that was established with White minority privilege as both a legal and customary norm. Serfontein’s and Betty’s lifetime torments, superimposed on fear of retaliation, create further adversity, impacting their health in the form of dementia. This element serves as a shackle that binds them to the past. The past is reframed as a symbol of an idyllic society, including cherished memories. Folded into these narratives that represent testimonies of the spirit of endeavor are the lengthy conversations between daughters and mothers. Thus, the narratives make room for heartfelt testimonies between people of different generations that reflect different ideological focuses, leading to deep and diverse reflections on the current state of South Africa.

In order to approach the aforementioned thematic elements, I employ the theoretical frameworks of life writing, aging studies, and, to a lesser extent, historiography. Thus, I explore the healing power of life narratives at large and the desire of people living with dementia to be vocal about the fears that take hold of them. The articulation of their experiences allows Serfontein and Betty to speak for themselves with a degree of authorial intervention, as their voices have a strong presence in the narrative. This will showcase the power of illness to unlock memories and create a repository of life, bringing to focus each of the mother’s history and personal narrative to maintain dignity and connection with their social and political *milieu* in later life. Relatedly, Serfontein and Betty operate as purveyors of an old culture in South Africa that can be accessed in the current era through the memorialization of their lived experiences. This key element evinces the cumulative impact of conflict throughout the lifespan in later life. This component stirs up past wounds, establishing a link with the compounding damage caused by the lack of processing stored-up emotions, the legacies of violence resulting from long-running disputes between social groups in the making of present-day South Africa, and apartheid injustices, which stoke tensions that persist in the present. As attested in the narratives, particularly in *Blood’s Inner Rhyme*, the cumulative impact of conflict reactivates not only past fears and conflicts but also sparks connection by developing a narrative self, thus proving right the idea that “narrative is the key to a self-examined, responsible life [that] makes possible an ethical relationship to the other” (Meretoja, 2018, p. 89). The narrative construction of such an ethical relationship between a daughter and a mother facilitates meaning-making and helps integrate the lived experience of Serfontein and Betty into a wider worldview.

The combined areas of expertise foster a key understanding and interpretation of past events while simultaneously promoting both alternative and community readings of contemporary South Africa among the readership that is fueled by the idea that illness opens doors (Charon, 2006, p. 177). Eventually, these narratives also constitute an archive for the study of the non-biomedical aspects of dementia in South Africa and elsewhere. Additionally, in this article I view dementia as a living archive that is built upon scraps of memory, thus reading people living with dementia not as empty vessels or as people who have lost their rational thinking (Behuniak, 2011) but rather as the custodians of repositories of personal and national history/historiography. This repository, even if it is a fragmented one, constitutes a living and dynamic archive, fostering connection between family members and providing insightful views into nation-building.

## Literature review: dementia studies and South African literature

The intersection of South African literary and cultural studies and dementia critical studies evinces that there is a critical gap in this emerging field of study in the southern tip of Africa. Currently, this gap is being addressed by some literary scholars, such as Hedley Twidle and the author, whose critical work bridges the fields of health humanities, aging studies, and literature within the frame of post-apartheid South Africa. These scholars aim to navigate the complexities surrounding memory loss, moving further by linking together the legacies of apartheid with the social, cultural, historical, and political singularities of South Africa.

To this end, there is a growing body of literary works (mostly memoirs) that deal with the realities of aging in South Africa. For instance, narratives that address this topical issue include Antjie Krog’s *Blood’s Inner Rhyme* (2025), Hedley Twidle’s “A Line of Light” in *Show Me the Place* (2024), Heather Ferris’ *Dementia and Our Love* (2024), Casey Golomski’s *God’s Waiting Room* (2024), Leslie Swartz’s *How I Lost My Mother* (2021), Colleen Higgs’ *My Mother, My Madness* (2020), Bridget Krone’s *Small Mercies* (2020), Julia Martin’s *The Blackridge House* (2019), Gerda Saunders’ *Memory’s Last Breath* (2017), Finuala Dowling’s *Homemaking for the Down-at-Heart* (2011), and in the passing, though, Liz McGregor’s *Khabzela* (2005). All these works situate dementia narratives within the framework of aging studies on South African soil, validating the assumption that the demographic shift in the context of South Africa is a tangible reality that is making its way to the front.

Yet, the production of critical thinking is still, slowly but surely, slipping into the literary and scholarly agendas, placing dementia at the forefront of the emerging field of literary gerontology in South Africa. Dementia critical studies in South Africa are therefore no longer a desert. It is being irrigated, and the terrain is, subsequently, blooming accordingly. In this regard, Twidle and Ortega-Montero’s work shifts away from Western interpretations of dementia, taking the enquiry beyond prosperous societies and understanding dementia care as a communal responsibility. The present paper represents an opportunity to revisit the aftermaths of apartheid through the critical lens of aging studies and literary gerontology, making novel interventions in the field.

This article is situated within the larger framework of dementia narratives in the Global South, with a focus on South Africa. Within cultural responses to what has been defined as the encounter between dementia, memory, and trauma (Krüger-Fürhoff et al., 2021), this research also sits in interdisciplinary perspectives that challenge the biomedical (and sometimes dehumanizing) and Euro-American frameworks of dementia critical and literary studies. Against this backdrop, my engagement with Krog’s and Martin’s dementia narratives, as I label them, explores how, paradoxically, in the context of forgetting, dementia narratives promote forms of untimely remembering in a post-conflict setting, as is the case in South Africa. Therefore, this paper considers the ways in which individual memory refracts and inflects community histories, embracing the possibility of dementia as a stand-in for national trauma.

## Memory and dementia in post-conflict South Africa

In *Blood’s Inner Rhyme* and *The Blackridge House*, dementia operates as a catalyst and plot device mechanism. Krog and Martin utilize non-linear and fragmented narratives, illustrating the workings of dementia as a living archive, to showcase how past historical conflict resurfaces across generations. In particular, such is the case when Serfontein’s and Betty’s memories start to fail, and their daughters step in. This fact initiates the transformation of their relationship, encompassing the reversal of roles and a significant plot point in their perception of the outer world. Despite significant ideological differences, particularly in the relationship between Serfontein and Krog, where political leanings act as a barrier, the two eventually develop a framework of mutual understanding. Whereas Serfontein is reluctant to political change, Krog celebrates it. Martin and Betty, in turn, learn about the nature of their bond by delving deeper into Betty’s lineage, where the political is not overly ideological.

It is through the exploration of family bonds and the significance of cultural legacies in the context of dementia that the scars of the past re-emerge, sometimes even revealing suppressed aspects of family history that are illustrative of the hardships of the struggle for control and ownership over economic resources in South Africa. This is particularly the case of *The Blackridge House*, when Martin “opened [a] suitcase [that] looked like an archive” (2019, p. 24). The suitcase contains diaries, photographs, and sentimental items that function as scraps of personal memory and national history, enabling Martin to navigate through her mother’s lineage. Martin is thus able to make connections across generations via sentimental items that address the gains and losses in Betty’s lifetime, namely the long-running dispute between the English settlers and Afrikaners in South Africa, which stems from episodes of violence in the making of South Africa. The archive, with memories, diaries, and photographs, intertwines sources that are essential for the reconstruction and reinterpretation of past cultures and political systems of South Africa, including the family’s involvement with the power structures of those systems in the colonial era.

In South Africa, a new political system has seen light in the space of a generation, which separates Serfontein and Betty from their daughters. It is a generation largely separated by cultural and political divides, where the older generation is the result of the aftermath of colonial contestation and the enforcement of tight social, cultural, political, and economic structures in the decades prior to and during apartheid. The younger generation, instead, bears the scars of that troubled past marked by war and racism-based trauma as part of the oppressor or beneficiary group, regardless of whether the families supported it or not. The narratives are instrumental in illustrating the anxieties of the former ruling class, functioning as historical archives, and examining to what extent holding a power position shaped their perception of apartheid South Africa. Although the lived experience and contestation of the oppressed class is only shown in passing in both narratives, it is important that both sides of the conflict operate as sources of historical truth. *Blood’s Inner Rhyme* and *The Blackridge House* can be interpreted as valid testimonies of an obscure chapter in the history of South Africa due to the portrayal of the inner workings of the White machinery, showing both how violence was orchestrated and the refusal to take on the role of the oppressor.

The scars of the past also include the different crises that still linger in the present, such as the construction of a democratic culture and the narratives that capture to examine the foundations of present-day South Africa. It is against this backdrop that Serfontein’s and Betty’s voices gain ground in the narrative in order to fill up the vacuum left by traditional representations of dementia, where the absence of the lived experience makes room for the speculation of meaning (Schou-Juul et al., 2026). In the life narratives, lived experience informs meaning-making as an active process on the part of Serfontein and Betty that is both shaped by their personal experiences and social contexts (Bormans & Van Gorp, 2025). This latter component underscores the intersection of dementia-induced forgetting and the apartheid era, which together form a multifaceted web of sociocultural and health challenges that urge resolution. Scholars of dementia critical studies have contended that people with dementia re-experience fears and violence of the past, resulting in flashbacks that compel them to revisit a vivid past (Gilmore-Bykovskyi et al., 2022; O’Rourke et al., 2016; Stončikaitė & Ortega-Montero, 2024). According to psychiatrist-anthropologist Helena Hansen, this behavior falls under the category of “diagnostic apartheid” (2019, p. 73).

Serfontein and Betty are haunted by a regretful past in myriad ways. First, it is necessary to consider the representation of Afrikaner culture, which plays an essential role in Krog’s memorialization of her mother. Serfontein operates as a cultural emblem of Afrikaner heritage and identity, which once conveyed a sense of power and is dwindling away in the present. Serfontein operates as the custodian of family identity and a fierce defender of Afrikaners’ contribution to South Africa as the former leading social group. In Serfontein’s eyes, “Afrikaners were a small nation that only wanted to do the best by everyone but became the victims of terrible propaganda” (2025, p. 53), and therefore, “Afrikaners’ plans [didn’t] quite work out” (2025, p. 55). Since its early inception, South Africa has been a place of contested boundaries, thriving in situations filled with conflict, misunderstanding, and outrage. Serfontein is trapped in that historical period, staking claims on Afrikaners’ right to stay in the country. Despite being increasingly trapped in a fog of forgetting, early-onset dementia brings back memories of suffering that appear to be deeply seated in her mind, operating as a stand-in for Afrikaner trauma. In her bid to work away the humiliations of the Boer War and the idea that Afrikaners were the only perpetrators of apartheid, Serfontein refuses the victimization of Afrikaners by saying that “as Black victims destroy each other and mess up the country’s affairs, the Afrikaner is feeling less and less guilty and becomes again useful in the creation of opportunities for himself” (2025, p. 102).

In *The Blackridge House*, in turn, instances of those historical memories and the creation of opportunities for the South African English come to the surface when Martin, as was said earlier, opens an old suitcase that reveals in vivid detail some silenced aspects of Betty’s infancy. The narrative thus locates readers in the midst of dispute over land with Black indigenous groups in the first decades of the twentieth century:

Those years of my mother’s early childhood, when her father had a decent job, a decade or so after what he describes as “the consummation of the Union,” were a time when it had already become more important for a certain class of colonials to be “White” than to be Boer or Brit. (2019, p. 49)

The early political construction of South Africa involved the creation of the so-called Union of South Africa (Braun, 2015), which united the White colonies of Transvaal, Orange Free State, the Cape Colony, and Natal—where parts of *The Blackridge House* take place. This act of “union” excluded Black people, leading to further conflict and giving rise to the present-day majority partner in the current ruling coalition in South Africa, the ANC, in its bid to counteract Afrikaner nationalism and the increasingly discriminatory attitudes that created a stifling atmosphere in the social and political terrains. The love story between Betty’s parents that is encapsulated in the narrative, and with which Martin was not fully familiar, cannot conceal the fact that despite being a pleasant land, with a dense ecology of trees, animals, and rivers, South Africa that was already in making had a lot of tensions underneath. Betty retreated into silence, and by making this narrative public, which Betty never shared openly with Martin, Betty reveals a great deal of sadness resulting from past historical armed conflict between Boers and Brits, but paving the way for a social order based on racial hierarchies:

A yellowing cutting from the *Natal Advertiser* of 1936 shows a small group of aging White men in suits gathered convivially around a big cannon. They are generals, colonels, and senators, among them Deneys Reitz and GMJ Molyneux, and, at the center, chatting, a thin but upright Major AW Smallie. “Only the Gun Was Neutral!” the headline declares, since this particular gun was used on both sides during the South African War. The tone is upbeat. For the very same weapon that these very men might well have used to destroy one another a generation before had become the instrument around which they could now stand together, smiling, in the redemptive postures of a new supremacy. (2019, p. 51, emphasis in the original)

*Blood’s Inner Rhyme* and *The Blackridge House* are instrumental in reflecting the importance of intergenerational, cultural, and political memory. The effects of historical conflict are regarded collectively, even in the absence of direct connection to some of the events on the part of the writers. Krog and Martin operate as the recipients of intergenerational trauma in various ways. Whereas Krog tries to overcome the idea that Afrikaner identity was fundamentally built in defeat, Martin approaches the inherited trauma resulting from the colonial construction of South Africa, highlighting the aspects that bring different social groups together. Political memory is thus underscored as one of the identity markers that shape the perception of the self in the context of dementia.

In the field of literary gerontology, there is a growing number of scholars (Nguyen et al., 2026; Roberto & Savla, 2025; Rolbieckie et al., 2026) who critically examine the ways in which dementia affects the family structures that support individuals with this condition and the perception of the self. In this sense, aging studies and life writing scholar Gill Constable (2013) contends that “[l]iving alongside a person with dementia helps us to develop knowledge about what is really important in people’s lives, including our own, and what gives joy and contentment” (p. 1). Thus, life narratives underscore the significance of maintaining a sense of identity as well as the human cost of cognitive decline by highlighting not only the disturbing nature of dementia but also those aspects that humanize this condition.

Life narratives engage with this question through the portrayal of identity fragmentation, which functions as a relevant theme in dementia-related life narratives (Kremmel, 2026; Stoncikaite & Ortega-Montero, 2024). Therefore, Krog and Martin convincingly demonstrate that their endeavors to preserve the core components of their mothers’ narrative identities are not futile, despite their difficulties in event sequencing. This component is relevant, as it assists Krog and Martin in their conceptualization of the self, thereby promoting cultural continuity. As pointed out earlier, Serfontein’s and Betty’s voices have a strong presence in the narrative. This element enables Krog and Martin to further explore forms of collaborative writing that evoke both fond and cherished memories. The ongoing sustenance of their sense of identity is best illustrated by the writers’ integration of conversational storytelling and relational practices.

Furthermore, the use of fragmentation in the narratives, in general, goes beyond the traditional view of the so-called untrustworthy narrators to compel the reader into the position of a vulnerable observer (Behar, 1996, pp. 1–33). Fragmented and non-linear accounts of those narratives provide a more nuanced understanding of the strategies writers come up with in order to recreate the lived experiences of their mothers and the multifaceted nature of their accounts (Strawson, 2004; Woods, 2011). Krog and Martin embody the role of vulnerable observers in their respective accounts of their mothers’ experience with cognitive decline. This aspect illustrates the ethical demands of storytelling, where intimate stories and historical events mingle together.

Additionally, this approach ensures that the narratives promote and validate the core principles of dignity and truthfulness, giving prominence to the well-being of all the parties involved in storytelling (Schultermandl, 2023; Turki & Ashmawy, 2024). This element evinces the power of literature as a culturally sensitive and inclusive tool in today’s societies. The narratives thus act as reminders of the need to prevent the repetition of the past. They also alert readers about the imperative need to preserve and further build the hard-earned democracy in South Africa, as the advent and consolidation of democracy ended needless death, at large, and ongoing conflict between social groups that were keen to spread their roots across the land. Those moments of intimacy between mothers and daughters, regardless of their positioning, put in value to the current age where equality is enforced.

Yet, intergenerational dialogue also serves as an outlet for Serfontein’s and Betty’s suppressed emotions to see light. It is only through that dialogue that the wounds of a tumultuous past start to heal. To this end, the lived experiences of Serfontein and Betty are faced with the reality of contemporary South Africa, a nation that continues to build up values and beliefs that are in stark contrast with the past embodied and experienced by the mothers. As a result, Serfontein, in particular, and Betty barely identify themselves with the workings of today’s South Africa. One such case is the discussion between Serfontein and Krog on the use of food resources or on the questionable legacy of Hendrik Verwoerd, whom Serfontein admired. Serfontein thus expresses her disappointment with Krog’s remarks that she could buy at the supermarket all the food she bottled for winter:

I know, but I can’t bear things so bountifully ripe when one doesn’t *do* anything with it… that’s the problem with you, with this whole country, you don’t know how to use what is actually there… You know they want to expropriate our farm, hey? (2025, p. 45, emphasis in the original)

Hendrik Verwoerd’s passing away is recorded in *Blood’s Inner Rhyme* as essentially the point at which, in the eyes of Serfontein, South Africa began to gradually but steadily succumb to dust and decay:

And as long as I live, I will never forget that Thursday morning when I was far from the house alone in the veld […] Elandspruit lies on the route taken by north-south aeroplanes, and we don’t notice them anymore. But suddenly I became aware of a droning noise from the southwest […] I looked up and suddenly recalled that the radio said the coffin with the remains of the Prime Minister would be flown to Pretoria today. Might this be it? I looked again. But no, I thought, it was only one aeroplane. There ought to be a whole squadron of our most beautiful, noblest aerial giants to take this man to his last resting place. (2025, p. 63)

Serfontein is aware of the backlash she might receive from Krog, who despises her mother’s evident “Verwoerdphilia.” Krog’s denial of any credit to the society that was founded upon the moral debasement of the majority of the population and corruption akin to that which inflicted destruction upon the Black people of South Africa becomes a source of conflict between mother and daughter. Their disagreement is exemplified by the use of racially derogatory language as a means of humiliation:

My and my mother’s politics had never gone together. Years ago, when I was still a young, fiery housewife here in Kroonstad, she used the k-word, which in those days was commonly used.

“I don’t want to hear that word in my house, ever,” I said to her.

She lost her temper. “Well, if I am not welcome in your house when I speak the way I speak, we essentially have nothing more to say to each other,” she shot back and stormed out.

Very early next morning […] [s]he swallowed. “I spent a long time thinking. You are right. This is your house, and you decide how you want in here and how people must behave. I will respect that. But then I expect that you also accept my choices in my house.” (2025, pp. 45–46)

In reality, Serfontein’s racism is a learned behavior. As the narrative emphasizes, her demeanor belies a childhood in which duty was always prioritized, and love was, if at all, a distant second. Largely speaking, as attested in the story, Serfontein’s incapacity to openly show affection characterizes a child whose unmet needs in a big family shaped both her personality and political views:

Well, the blind embrace of apartheid, and sometimes I wonder whether the mighty amounts of food that are dished up in Afrikaner houses is a leftover of starvation…Perhaps also the absence of physical contact. When you’ve buried so many children, maybe you cannot bring yourself to show affection to a child. I can’t remember a single time that my father touched me the way you young people tickle your children or kiss and caress. No. But by his deeds, what he did for me, how fully he was intent on his doings, that is how I know of his love. (2025, p. 59)

Krog’s family history contrasts with that of Martin, where family relations evoke love and affective experiences, even in the context of competition with Black social groups in the then newly emerging colonial system. As Martin recalls, Blackridge was “a living world in which everyone was together, assembled, and at home” (2019, p. 151). Martin brings into focus how the imaginary of family life is imbued with peace and tolerance, highlighting bonds between family members, the home environment, and nature. The memoir evinces a profound comprehension of human feelings and relationships, despite the fact that the portrayal of conflict played a central role in colonial South Africa. The narratives are unequivocal in their assertion that dementia did not diminish the personhood of either Betty or Serfontein.

The typical categorization of dementia narratives as either medical or personal accounts that walk readers into deep illness and are mediated for the sake of conveying meaning is defied by Serfontein’s and Betty’s stories (Burke, 2015, 2018; Zeilig, 2012, 2014). Moreover, narratives about dementia are rarely considered within the larger framework of national history (Krüger-Fürhoff et al., 2021). Serfontein’s and Betty’s vocalization of their experiences in *Blood’s Inner Rhyme* and *The Blackridge House* contravenes this trend, offering an alternative reading of past conflicts, namely: colonial legacy and apartheid, which are fundamental to the current state of South African society. Moreover, *Blood’s Inner Rhyme* and *The Blackridge House* challenge the medicalized view of dementia in order to give prominence to “storied selves,” blurring the lines between dementia care practices and personal/collective history. This element underscores that no matter who lives through the past or performs past actions, memories can actually mirror community consciousness.

The significance of dementia gains ground in the critical exploration of narratives that deal with key historical events, employing the emotional implications that dementia entails in order to navigate through some of the unpleasant aspects of South African history. Reimagining the past in the present is a challenging task due to the extensive shadow it casts. As Krog notes:

Our historical novels that take place against the background of the occurrences that so sweepingly influenced the existence of our nation reveal that those who dared it have a great shortcoming. The writers are seldom able to bring across the deeper linkage between man and his past in an epic and convincing manner. (2025, p. 83)

In her critique of writers’ failure to comprehend the historical events that shaped South Africa, Serfontein fails to recognize that Krog’s or Martin’s work is crucial to establishing a sense of cultural continuity between Serfontein and Betty and nation-building.

*Blood’s Inner Rhyme* and *The Blackridge House* may well read as historiographical narratives that provide personal reinterpretations of past events, somehow similar to how Serfontein or Betty constructed narratives from memory. The narratives, as will be dealt with in the section to come, feature lived experiences that grapple with historical legacy, cultural heritage, and/or the transmission of values. The latter is essential to understanding the construction of the mothers’ identity, validating the idea that memorialization makes distant events relatable. Memorialization thus contributes to meaning-making and the preservation of personhood in later life, confirming that human agency remains in constant evolution in the life span, even in the context of dementia.

## Narrative reconstruction in later life and the role of family archives

In aging and life writing studies, the narrative reconstruction of the self in later life is essential for the purpose of comprehending how individuals reshape their life stories and maintain a steady sense of the self (Eakin, 1999; Oró-Piqueras & Falcus, 2018). As is often the norm, this crucial process takes place amidst age-related conditions that invite individuals to review their identities across their lifespan. Such a process usually involves integrating the past and present into a meaningful narrative that brings together life events of all sorts, particularly, but not exclusively, revisiting both disruptive moments and turning points in life, into a larger, more comprehensible, and easily accessible life narrative. Furthermore, in this meaningful process, family archives are crucial for rebuilding their lives, thus finding purpose as well as shaping the mothers’ fragmented lives into a more cohesive narrative in old age.

Scholars in aging studies and literary gerontology tend to read old people as wells of wisdom (Ardelt & Edwards, 2016; Ortega-Montero, 2025; Stončikaitė, 2022; Stončikaitė & Ortega-Montero, 2024; Švaríčková-Slabáková, 2022), and this positioning creates the ideal breeding ground for old people and individuals around them to revisit experiences and potentially unresolved issues or events that continue to haunt them in later life. In the life narratives under analysis, the exploration of uncharted territories is essential for Krog and Martin to gain knowledge about the longings and desires of their mothers, revealing not only the experiences that they embrace and/or reject but also the vital processes that have shaped their cosmovision. This aspect provides opportunities to strengthen the bond between mothers and daughters, thus promoting female affiliation, as is often the case in narratives written by women (Ortega-Montero, 2025; Zygadlo, 2012). In the narratives, female affiliation takes the form of collaborative writing through conversational storytelling.

Life writing scholar Lars-Christer Hyden (2018) considers that the so-called collaborative dementia life narratives offer insights into the fabric of narrative identity in later life, showcasing how the cultivation of healthy bonds in later life helps people with dementia keep grounded and secure. Relatedly, aging studies and life writing scholar Rebecca Bitenc contends that in the context of collaborative dementia life narratives, the primary task of the writer is basically to produce a meaningful and coherent narrative that preserves the notion of normalcy in the face of memory loss (2020, pp. 99–100). This component is rendered crucial for building a narrative environment conducive to mutual respect and understanding, even if ideological differences act as a barrier at some point in their relationship. Life writing scholar Paul John Eakin deepens the notion of understanding when he contends that life narratives, besides representing other people’s lives, are also tools of self-representation and self-understanding (2006, p. 184). This is a core idea that illustrates Krog’s and Martin’s engagement with the narrative identity of their mothers because, as Eakin argues, “the extended self is the self of memory” (2020, p. 2), and such a thing builds stronger bonds between mothers and daughters.

In *Blood’s Inner Rhyme* and *The Blackridge House*, Krog and Martin create narratives of acceptance in the sense that both Serfontein and Betty face the irreversible constraints of aging in the form of gradual cognitive decline. This matter-of-life fact compels the authors to come to terms with life itself as it comes, placing narrative emphasis on the pursuit of personal realization and navigating the complexities surrounding dementia as best as is possible for them. In so doing, Krog and Martin integrate discordant events of the past into the construction of the narrative identities of their mothers. In this regard, life narratives are the preferred genre for a deep dive into the examination of narrative identities in the context of illness (Birren & Deutchman, 1991; Couser, 2021; Hyden & Orulv, 2009; Walker, 2023). Discordant aspects of the past particularly affect the relationship between Krog and Serfontein. In their case, acceptance revolves around sustaining their interpersonal bond in the face of persistent and insurmountable ideological constraints that, more often than not, prevent Krog and Serfontein from fully expressing their mutual love and profound admiration.

In the first case under analysis in this section, *Blood’s Inner Rhyme*, the narrative often provides a portrait of Serfontein as a social misfit in post-apartheid South Africa as well as a taciturn and opinionated woman who feels alienated from the society she inhabits as a result of racial and perceived national differences. Thus, Krog identifies the central conundrum in Serfontein’s life to be nostalgia. Serfontein’s inability to face adversity in democratic South Africa compels her to retreat into her own microcosm, often detached from reality and inhabiting a space where, in her view, failures outnumber successes.

This feeling is further exacerbated by the fact that in post-apartheid South Africa Serfontein sees her former power as part of the White minority ruling class dwindling, especially in the context of frail care where Serfontein is dependent on the formerly disenfranchised: the Black South African women who perform the duties of caregivers. In the narrative, this view comes to the fore through Victoria, Serfontein’s Black caregiver. As Victoria notes:

To have a bond with white people is more difficult than having a bond with old black people. Black patients are anxious, and you have to reassure them–that is the task. While white patients…they come from the era where they had complete control, they were the lords of everything, of themselves and over everybody else. So, they want to dominate, they are aggressive, and to change that relationship to a new relationship between a white baas and a black carer…that is complicated. (Krog, 2025, pp. 211–212)

Facing the realities of aging in democratic South Africa forces Serfontein to recalibrate, as she is now somehow living on the edge of comfort. In this sense, it is important to note, as critical voices in the field of narrative identity argue, that her personal challenge is “to accept adversity as part and parcel of life, to endeavor to manage adversity when it cannot be overcome” (McAdams et al., 2022, p. 5). Consequently, coping with the intricacies of old age remains a central concern for Serfontein, who is trapped by some darkness and who is herself feeling vulnerable. As Krog recalls:

She stood like that for a long time, then almost sobbed out a sigh and came to sit slowly in her chair. Together we listened to the vulnerability and longing in that mighty voice.

“What has happened to that part of me who listened to this with rapture? The worst about getting old is that everything is so dead. Dead. Somewhere inside you, now and then, faded memories may stir, reminding you that once, upon my soul, once you *felt*!” (2025, pp. 57–58, emphasis in the original)

Serfontein’s feeling of deadness stems from her awareness of mortality, which, due to old age, is looming on the horizon. The aging process often gives rise to feelings of vulnerability due to loss of independence and social isolation. Serfontein’s narrative identity struggles to navigate vulnerability in aging.

The examination of vulnerability in aging in *Blood’s Inner Rhyme* not only serves as a central identity marker in old age but also fosters connection and helps build resilience, thus practicing gratitude and cultivating positive aging attitudes in the narrative. This is of utmost importance, as it helps push deep existential vulnerability to the margins in favor of an ablest perception of aging, particularly one that does not view it as a burden. Care ethics scholar Virginia Held pushes in this direction when she locates the importance of care within the frame of a global culture of neglect, placing emphasis on relational practices as a vehicle to counteract vulnerability (2006, p. 165). In this sense, Judith Butler provides one engrossing and pertinent definition of vulnerability that can be applied to *Blood’s Inner Rhyme*:

We come into the world unknowing and dependent, and, to a certain degree, we remain that way. We can try, from the point of view of autonomy, to argue with this situation, but we are perhaps foolish, if not dangerous, when we do. Of course, we can say that for some this primary scene is extraordinary, loving, and receptive, a warm tissue of relations that support and nurture life in its infancy. For others, this is, however, a scene of abandonment or violence or starvation; they are bodies given over to nothing, or to brutality, or to no sustenance. No matter what the valence of this scene is, however, the fact remains that infancy constitutes a necessary dependency, one that we never fully leave behind. (2004, pp. 23–24)

In the eyes of Butler, all humans are, in various degrees, inherently vulnerable. Serfontein’s vulnerability mostly stems from her perceived dwindling authority on family and social levels. This helps to illustrate that, in her eyes, the social system in post-apartheid South Africa amplifies her perception of becoming a vulnerable individual, making her life grievable in later life, as it is shaped by norms she tolerates but, simultaneously, greets with certain scorn and resistance. In essence, Krog’s narrative construction of her mother connects Serfontein’s frailty not only to dementia but also to the current politics of South Africa, which Serfontein openly disagrees with in the narrative, as was earlier discussed. Serfontein’s narrative identity is in stark contrast with that of Martin’s mother, Betty, who is presented as an individual who, in later life, seeks to revisit her childhood.

The construction of Betty’s narrative identity in old age revolves around two fundamental issues: family archives and attachment to land. In the context of South African life writing, archives play a key role in bringing to light silenced bits of history and suppressed aspects in the lives of individuals and communities, thereby rewriting parts of its troubled history through the lens of ordinary people (Coullie et al., 2006; Ngwenya, 2000; Nivesjö, 2022; Nuttall & Coetzee, 1998). Drawing on this core idea, Betty’s identity plays itself out, with Martin tracing family roots in her bid to understand not only Betty’s lineage but also hers. In *The Blackridge House*, everything starts out when Martin “could no longer ignore the old suitcase at the top of [her] mother’s cupboard in the nursing home” (2019, p. 24). Martin herself acknowledges that she never paid attention to the suitcase. However, the suitcase propels her into a quest full of surprises and discoveries because, as Martin admits, “[a] quest is never what you expect it to be” (2019, p. 23). It is actually a well-documented quest by which Martin solidifies the survival of Betty’s memories, passing them down not only to the next generation but also creating a valuable set of documentary tools, including the writing of the memoir, for a better comprehension of personal and family journeys. The old suitcase constitutes the raw material for Martin to build the narrative identity of Betty. This element is essential in the South African context, as the country still today grapples with developing a narrative about its fragmented past, thus highlighting once again the value of family archives in bridging the personal with the national.

Featured as a central element in *The Blackridge House*, together with Martin’s house search, which gives name to the life narrative, is the family archive. Criticism highlights “how family archives shape the formation of identity and the role the archive plays in marking the progression and form of the social life course” (Gloyn et al., 2018, p. 158). In line with this, Martin’s narration of Betty’s archive and construction of identity in later life constitutes a form of transparent historiography (Holtschneider & Shofar, 2019, p. 331), where attachment to land and the richness of nature showcase one of Betty’s central identity markers. As Martin notes:

By the time [Betty] was telling stories about Blackridge in the nursing home, it had become inhabited by events and people from many years hence–like the garden in a hermetic *Ars Memoriae*, which she’d populated with images compelling enough to guide her through memory when she returned to walk its paths. (2019, p. 31, emphasis in the original)

In the life narrative, the picturesque landscapes of Blackridge are narrated in detail, illustrating both Martin’s natural tendency for the representation of the environment, her field of study, and more importantly, Betty’s teachings of the importance of the preservation of the environment. As a result, Martin places great emphasis on this defining aspect of Betty’s personality, revealing Betty’s sensitivity to the awareness of the place. As Martin points out, “[she] realized that for [Betty] the experience of that primal ecosystem became, other memories faded, a quintessential emblem of the beautiful” (2019, p. 91).

Betty’s recollection of childhood memories in *The Blackridge House* also plays a crucial role in stimulating the recording of memories through both the vivid representation of the place and sensory engagement, particularly sounds and smells, which evoke positive emotions and help Betty reduce her levels of anxiety in the care home. As was shown in this section, Krog and Martin rely on different elements to construct their mothers’ narrative identity, recurring to their mothers’ self-defining aspects to convey the most important aspects of their lives, providing continuity to their lives, and emphasizing how Serfontein and Betty continue to be the custodians of family identity, even after their deaths. This example demonstrates the value of illness narratives to function as permanent living archives that surpass the limitations of life and, as some critical voices contend, extend beyond loss (Hyden et al., 2014).

## Conclusions

This article has examined *Blood’s Inner Rhyme* and *The Blackridge House* by approaching the stories through the critical lens of aging studies and life writing theory, incorporating elements from the field of health humanities and historiography into the analysis. Krog and Martin portray how Serfontein and Betty struggle with dementia, making their lived realities public and accessible. The representation of the social and cultural contexts of health in the narratives under scrutiny serves to illustrate the ever-increasing systematic evidence of how narrative approaches to health are both solidifying and slipping slowly but surely into the literary agenda.

This fact demonstrates that life writing and aging studies, as forms of humanities-based inquiry, contribute to a comprehensive analysis and understanding of the self. Moreover, they function as a valuable lens through which individuals can investigate the often intricate relationship between family members as well as the sociocultural dimensions of health, especially in later life. As a result, the narratives emphasize the significance of continuous learning and dignity in old age (Kuyler et al., 2023; Tauzer et al., 2023).

The analysis of these two life narratives also underscores how the very act of writing out experiences of illness provides safe spaces for intergenerational dialogue, which, in the case of South Africa, constitutes an act of healing. More specifically, analyzing stories of dementia reveals the profound impact that historical conflict has on older people. In spite of their deteriorating memories, old people revisit past events that are deeply ingrained in their emotional memories because, in the end, the heart always remembers. This article also contributes to the fundamental idea that a more informed society must be constructed in order to address new perspectives on aging and illness while maintaining respect for all segments of the population. In this regard, life narratives constitute vehicles for individuals and communities to find their place in society.

To conclude, Krog’s and Martin’s accounts of dementia are instrumental in creating meaning-making devices in the context of illness, thus creating solid bonds between a mother and a daughter. This illustrates the importance of networks of solidarity and care, showcasing the singularities of aging through the healing and therapeutic power of life writing. As Krog notes, “[y]our life is a big story” (2025, p. 232), and discussing it in the context of dementia transcends the conventional features of biomedical models, which tend to ignore the identity of individuals in favor of neuropathological factors that disregard real-life experiences (Kontos, 2003). While Krog and Martin also engage the readers in a hands-on approach to illustrate the complexities of South African history, including the adversities that South Africans faced over time, both life narratives also express the aspirations and longings of two bedridden women. Serfontein and Betty remain powerful and deserving until the very end of their lives, and this aspect is most laudable.

## Data Availability

This article does not report data, and therefore the pre-registration and data availability requirements are not applicable.
